# Comparative survey of go/no-go results to identify the inhibitory control ability change of Japanese children

**DOI:** 10.1186/1751-0759-8-14

**Published:** 2014-07-11

**Authors:** Koji Terasawa, Hisaaki Tabuchi, Hiroki Yanagisawa, Akitaka Yanagisawa, Kikunori Shinohara, Saiki Terasawa, Osamitsu Saijo, Takeo Masaki

**Affiliations:** 1 Shinshu University, Faculty of Education, 6-Ro Nishinagano Naganoshi, Nagano 380-8544, Japan; 2 Physical Fitness Research Institute, Meiji Yasuda Life Foundation of Health and Welfare, 150 Tobukimachi Hachoujisi, Tokyo192-0001, Japan; 3 Department of Infant Childcare, Matsumoto Junior College, 3118 Sasaga Matsumotosi, Nagano 399-0033, Japan; 4 Center of General Education and Humanities, Tokyo University of Science, 5000-1 Toyohira Chino, Nagano 391-213, Japan; 5 Department of Electrical and Electronic Engineering, Shinshu University, 4-17-1 Wakasato Naganoshi, Nagano 380-928 Japan; 6 Laboratory of Psychology, Nippon Sport Science University, 7-1-1 Fukasawa Setagayaku, Tokyo 158-0081, Japan

## Abstract

This research, conducted in 1998 and 2008, uses go/no-go data to investigate the fundamentals of cognitive functioning in the inhibitory control ability of Japanese children. 844 subjects from kindergarten to junior high school participated in go/no-go task experiments. Performance of go/no-go tasks, which are frequently used to investigate response inhibition, measures a variety of cognitive components besides response inhibition. With normal brain development, the ability to inhibit responses improves substantially in adolescence. An increase over time in the error rate during the go/no-go tasks of subjects of the same age indicates that these processes are not functioning properly. Comparisons between the 1998 and 2008 data revealed several differences in error rates. In 2008, there were increases in the number of errors in groups from each age range. The comparison also revealed that overall error rates peaked at later ages in the 2008 subjects. Taken together, these results show changing conditions in the inhibitory function of the prefrontal cortex. However, the reason for these changing conditions remains unclear. While a lifestyle questionnaire revealed several differences in factors such as bedtimes and hours spent watching TV, analysis did not reveal a significant correlation.

## Introduction

Cognitive control functions continue to improve from infancy until early adulthood, allowing flexible adaptation to a complex environment [1]. Growth in executive functioning skills play a role in children's academic success, and the transition to elementary school is an important time for the development of these abilities [2]. Executive functions make it possible to mentally play with ideas and take the time to think before acting. Core executive functions are inhibition and interference control, working memory, and cognitive flexibility [3]. Both inhibitory-based executive functioning and basic information processing deficits are found in clinic-referred attention deficit hyperactivity disorder samples [4].

Response inhibition is an essential executive function implemented by the prefrontal cortex.

Recent neuropsychological and neuroimaging studies have shown that the presupplementary motor area and ventrolateral prefrontal cortex are crucial for response inhibition and that various subregions of the prefrontal cortex make different contributions leading to successful response inhibition [5]. Performance of go/no-go tasks, which are frequently used to investigate response inhibition, requires a variety of cognitive components besides response inhibition [6].

In view of the changing cultural conditions and their potential influence on response inhibitory function implemented by the prefrontal cortex, we have been investigating the condition of children’s inhibitory function. In particular, we have been focusing on the inhibitory control ability using go/no-go tasks since 1969 [7-10]. The number of errors made by sixth and seventh graders in go/no-go tasks has been increasing since around 1980.

Despite this, no research has applied go/no-go tasks to subjects from kindergarten to junior high school to explore the process of inhibitory development. Thus, we investigated the developmental inhibitory process of the repression function of children from kindergarten to junior high school using go/no-go tasks.

## Methods

### Participants

The participants were children aged 3 to 15 years from the same kindergarten-to-junior high school (G1 to G12). 437 children (225 boys and 212 girls) participated in 1998 and 407 different children (200 boys and 207 girls) participated in 2008 (see Table 1 for details). In this investigation, no children with ADHD or autism were included. The latest guidelines of the Helsinki Declaration were followed, and the study was approved by the Institutional Ethics Committee of Shinshu University. Written informed consent was obtained from the children and their parents.

**Table 1:** The number of participants

**Grade level**	**Age (years)**	**1998**	**2008**
G1	3 - 4	23	28
G2	4 - 5	53	46
G3	5 - 6	53	30
G4	6 - 7	37	33
G5	7 - 8	37	35
G6	8 - 9	32	28
G7	9 - 10	35	29
G8	10 - 11	33	35
G9	11 - 12	32	31
G10	12 - 13	36	38
G11	13 - 14	31	38
G12	14 - 15	35	36

### Task

**Figure 1:**
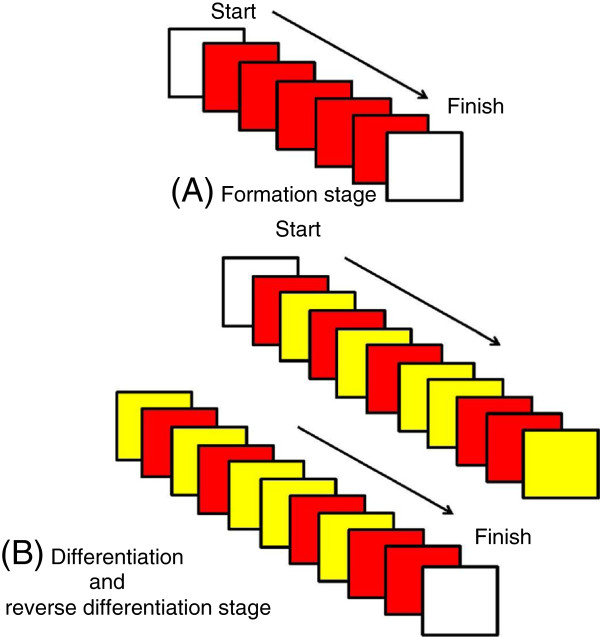
**The presentation order of Go/No-Go tasks. (A)**: During the formation stage, a red light was displayed to train participants in when to grasp a rubber ball. **(B)**: The stimuli were the same for the differentiation and reverse differentiation stages. During the differentiation stage, participants were asked to squeeze the rubber ball when the light was red, but not when it was yellow. The roles of the red and yellow lights were reversed during the reverse differentiation stage.

The go/no-go task consisted of the following three stages: formation, differentiation, and reverse differentiation. In the formation stage, the participants were trained to squeeze a rubber ball in response to a red light stimulus, which was displayed five times (Figure 1A). For the differentiation stage, they were asked to squeeze it in response to red but not to yellow (Figure 1B). For the reverse differentiation stage, the instructions were reversed; that is, they squeezed the ball in response to yellow but not to red. Each participant performed 20 trials each during the differentiation and reverse differentiation stages and the number of errors from the 40 trials of the two stages was calculated. Yellow and red stimuli were used for G1 to G9 subjects, and bright and dim lights were used as stimuli for G10 to G12 subjects. The duration of each stimulus was random between 200 and 1100 ms. The inter-stimulus interval was also random between 1300 and 7500 ms.

The participants were seated in a booth enclosed on three sides by 45 × 45 cm panels. Stimuli were presented in a framed rectangle (2.5 cm × 3.7 cm), which was approximately 70 cm away from their eyes. The experiment was conducted by computer-controlled equipment (ME Corporation, Nagano, Japan).

### Statistical analysis

We used the number of errors for statistical analysis of the go/no-go tasks. Reaction times could not be determined because we used analog data collection in 1998. In this study, we analyzed the data classified according to age as defined by educational grade. Preliminary analyses were conducted to determine if there were any differences in the number of errors made between each grade in kindergarten, elementary school and junior high school using one-way ANOVA and Tukey’s honest significant difference post hoc multiple comparison test. We used two-way ANOVA to make a comparison between 1998 and 2008 data. Because the data obtained from the go/no-go tasks have a limited number of errors and a normal distribution is not assumed in this case, secondary analyses used the Poisson distribution.

The participant’s wakeup time, bedtime, and time spent playing, studying, watching TV and playing videogames were assessed from a questionnaire filled out after the experiment. The results of the questionnaires from 1998 and 2008 were compared using independent *t*-tests. In addition, we investigated the correlation between the number of errors for the go/no-go task and the numerical values of the 2008 questionnaire.

The level of significance was set at *p <* 0.05. Statistical analyses were performed using SPSS Statistical Packages (SPSS Inc., Chicago, USA).

## Results

For the convenience of statistical analysis, the participants were categorized into three respective age groups: kindergarten (G1 to G3; ages 3 to 6 years), elementary (G4 to G9; ages 6 to 12 years), and junior high school (G10 to G12; ages 12 to 15 years, see Table 1 for details).

### The number of errors in 1998

**Figure 2:**
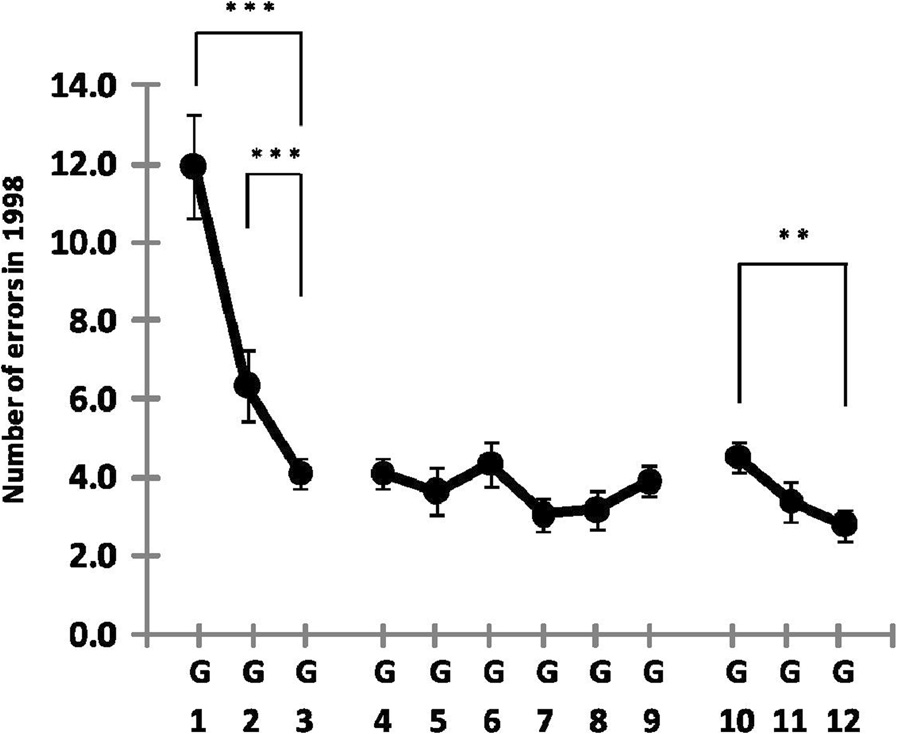
**Number of errors for G1 to G12 in 1998.** The data points and error bars indicate mean and the standard error, respectively. Significant differences are denoted with ** for *p <* 0.01 and *** for *p <* 0.001.

Figure 2 shows that the number of errors decreases with age, especially between G1 and G3. An independent ANOVA indicated that the effect of age group was significant [F(2,126) = 18.7, *p <* 0.001]. For the kindergarten group, Turkey’s HSD analyses showed that the numbers of errors for G2 (mean (M) =6.4, standard error (SE) =0.9) and G3 (M = 4.1, SE = 0.4) were significantly lower than for G1 (M = 12.0, SE = 1.3) [G1 vs. G2, *p <* 0.001; G1 vs. G3, *p <* 0.001]. The results of the elementary-school group from G4 to G9, however, were almost the same across these different ages [F(5,200) =1.1, *p >* 0.37]. In the junior-high-school group, the number of errors also decreased with age [F(2,99) = 27.4, *p <* 0.01]. The difference between G10 (M = 4.5, SE = 0.4) and G12 (M = 2.8, SE = 0.4) was significant [*p <* 0.01].

### The number of errors in 2008

**Figure 3:**
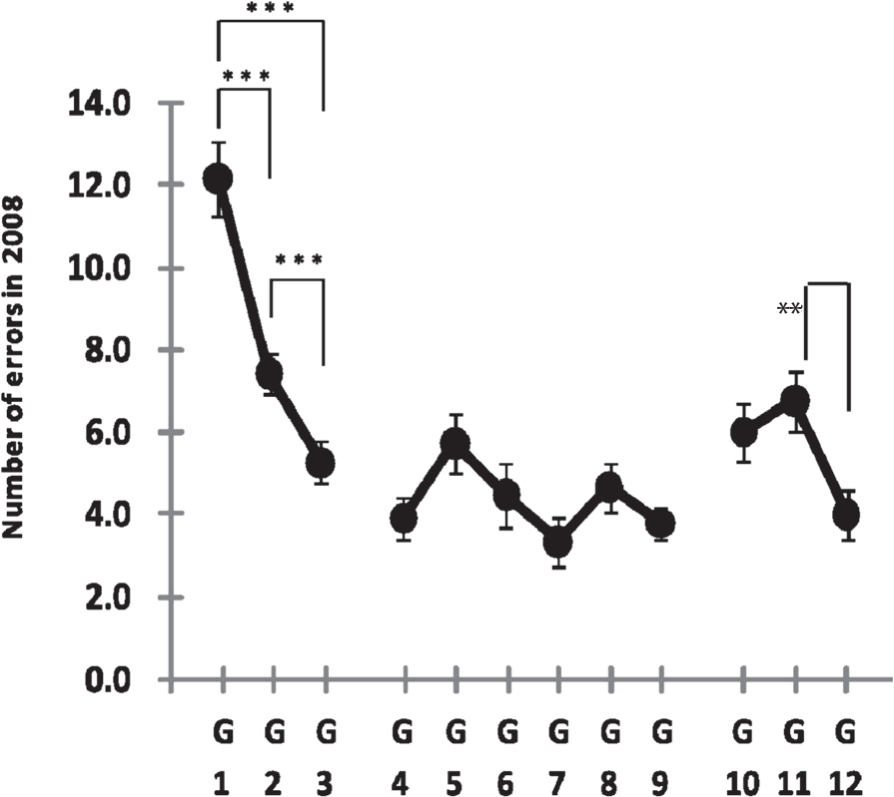
**Number of errors for G1 to G12 in 2008.** Significant differences are denoted with ** for *p <* 0.01 and *** for *p <* 0.001.

Figure 3 shows that the number of errors decreased with age from G1 to G3, similar to what was observed in 1998. An independent ANOVA indicated that the effect of age group was significant [F(2,101) = 24.0, p < 0.001]. The numbers of errors for G2 (M = 7.4, SE = 0.5) and G3 (M = 5.3, SE = 0.5) were significantly lower than for G1 (M = 12.1, SE = 0.9) [G1 vs. G2, p < 0.001; G1 vs. G3, p < 0.001]. The elementary-school group from G4 to G9 did not show a statistically significant difference in error rate [F(5,185) = 23.5, p > 0.09]. In the junior-high-school group, the difference in the number of errors between G11 (M = 6.8, SE = 0.7) and G12 (M = 4.0, SE = 0.6) was significant [p < 0.01].

### Comparison between 1998 and 2008

**Figure 4:**
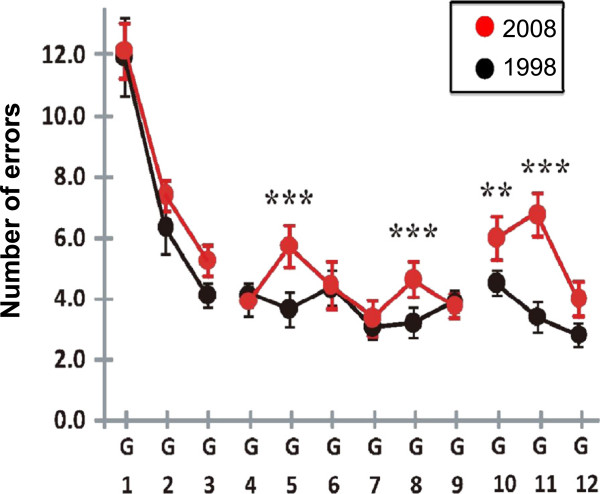
**Superimposed results for 1998 (black line) and 2008 (red line).** Significant differences are denoted with **: *p <* 0.01, ***: *p <* 0.001.

The results of 1998 and 2008 are superimposed in Figure 4. Although the overall patterns are similar, the numbers of errors in 2008 tended to be greater than those of 1998. The greatest difference for the different years of investigation was found in G11; the average number of erroneous responses was 3.4 in 1998, as opposed to 6.8 in 2008. The effect of the research year was statistically significant [F(1,198) = 14.7, p < 0.001]. A post-hoc analysis based on a Poisson distribution indicated that the 2008 results for G5, G8, G10, and G11 were statistically higher than those of 1998 [p < 0.001].

### Lifestyle survey and correlational investigation

Table 2 shows the mean response from each survey question in 1998 and 2008. Although some lifestyle questions, such as wakeup time for G4, bedtime for G5, and time spent studying for G12, were found to be statistically significant between the survey years, the pattern of significance seemed too sporadic to interpret.

**Table 2:** Results from the lifestyle questionnaire in 1998 and 2008

	**G-1**				**G-2**				**G-3**			
	**1998**	**2008**	***t***	***p***	**1998**	**2008**	***t***	***p***	**1998**	**2008**	***t***	***p***
Awake time	7:07	7:11	-0.632	NS	7:16	6:54	2.587	*	7:14	7:05	1.653	NS
Bedtime	21:12	21:01	0.943	NS	21:08	20:49	1.873	NS	21:09	20:36	1.194	NS
Time to play (min)	215	182	1.912	NS	227	186	3.187	**	201	155	2.790	**
Time to study (min)	-	-	-	-	-	-	-	-	-	-	-	-
Time to watch TV & play videogame	94	114	-1.203	NS	101	101	0.022	NS	79	87	-0.487	NS
	**G-4**				**G-5**				**G-6**			
	**1998**	**2008**	***t***	***p***	**1998**	**2008**	***t***	***p***	**1998**	**2008**	***t***	***p***
Awake time	6:35	6:19	3.455	***	6:30	6:17	0.126	NS	6:44	6:22	-1.223	NS
Bedtime	20:56	21:01	0.102	NS	20:54	21:18	-3.491	***	21:10	21:12	1.453	NS
Time to play (min)	95	71	2.025	*	87	73	1.156	NS	100	91	-1.210	NS
Time to study (min)	80	46	-0.138	NS	115	57	2.519	*	114	71	-0.648	NS
Time to watch TV & play videogame	86	83	2.077	*	133	98	3.194	**	124	174	-0.871	NS
	**G-7**				**G-8**				**G-9**			
	**1998**	**2008**	***t***	***p***	**1998**	**2008**	***t***	***p***	**1998**	**2008**	***t***	***p***
Awake time	6:32	6:20	0.987	NS	6:32	6:19	-1.408	NS	6:28	6:19	1.776	NS
Bedtime	21:10	21:32	0.958	NS	21:17	22:08	-2.626	**	21:32	21:56	-0.398	NS
Time to play (min)	75	75	-0.120	NS	85	63	0.690	NS	60	53	0.954	NS
Time to study (min)	158	89	0.544	NS	113	98	-1.498	NS	183	111	0.974	NS
Time to watch TV & play videogame	110	132	0.395	NS	125	114	1.283	NS	93	106	2.334	*
	**G-10**				**G-11**				**G-12**			
	**1998**	**2008**	***t***	***p***	**1998**	**2008**	***t***	***p***	**1998**	**2008**	***t***	***p***
Awake time	5:45	5:56	-1.336	NS	5:54	5:57	-1.427	NS	5:50	6:17	-2.593	*
Bedtime	23:02	22:29	2.790	**	23:18	23:17	0.054	NS	23:39	23:57	-1.227	NS
Time to play (min)	27	45	-1.982	NS	67	111	-2.658	**	27	132	-2.922	**
Time to study (min)	127	119	0.592	NS	105	113	-0.554	NS	218	158	4.168	***
Time to watch TV & play videogame (min)	99	95	0.246	NS	117	166	-1.460	NS	61	97	-2.804	**

NS, not significant; *p < 0.05; **p < 0.01; ***p < 0.001.

Table 3 indicates the correlation between each lifestyle question in 2008 and the number of errors for the go/no-go task. A positive correlation coefficient indicates that the number of errors increases as the numerical value for the survey question increases, whereas a negative correlation coefficient indicates that the number of errors decreases as the numerical value for the survey question increases. The error rate and time spent watching TV and playing videogames were positively correlated for G5 and G8, although the strength of relationship was moderate or less. A negative correlation was also found for time spent studying for G4 and G8.

**Table 3:** Correlations between number of errors and lifestyle questionnaire responses in 2008

	**G-1**			**G-2**			**G-3**		
	***r***	***t***	***p***	***r***	***t***	***p***	***r***	***t***	***p***
Awake time	0.117	0.003	NS	-0.132	-0.880	NS	0.188	0.994	NS
Bedtime	0.010	0.053	NS	-0.078	-0.522	NS	0.054	0.278	NS
Time to play (min)	-0.304	-1.630	NS	-0.060	-0.436	NS	-0.203	-1.078	NS
Time to study (min)	-	-	-	-	-	-	-	-	-
Time to watch TV & play videogame (min)	-0.144	-0.739	NS	-0.137	-0.914	NS	0.097	0.504	NS
	**G-4**			**G-5**			**G-6**		
	***r***	***t***	***p***	***r***	***t***	***p***	***r***	***t***	***p***
Awake time	-0.140	-0.746	NS	-0.121	-0.692	NS	0.297	1.583	NS
Bedtime	-0.214	-1.161	NS	0.098	0.556	NS	-0.212	-1.109	NS
Time to play (min)	0.032	0.172	NS	-0.024	-0.139	NS	0.198	1.027	NS
Time to study (min)	-0.375	-2.146	*	-0.306	-1.821	NS	0.015	0.077	NS
Time to watch TV & play videogame (min)	-0.188	-1.015	NS	0.443	2.796	**	0.002	0.010	NS
	**G-7**			**G-8**			**G-9**		
	***r***	***t***	***p***	***r***	***t***	***p***	***r***	***t***	***p***
Awake time	0.149	0.737	NS	0.045	0.256	NS	0.039	0.212	NS
Bedtime	-0.231	-1.161	NS	-0.168	-0.965	NS	0.181	0.989	NS
Time to play (min)	0.183	0.911	NS	0.049	0.277	NS	-0.072	-0.391	NS
Time to study (min)	-0.199	-0.992	NS	-0.405	-2.507	*	0.225	1.243	NS
Time to watch TV & play videogame (min)	-0.187	-0.933	NS	0.177	1.018	NS	-0.009	-0.050	NS
	**G-10**			**G-11**			**G-12**		
	***r***	***t***	***p***	***r***	***t***	***p***	***r***	***t***	***p***
Awake time	0.058	0.346	NS	-0.051	-0.308	NS	-0.030	-0.172	NS
Bedtime	0.103	0.624	NS	0.000	0.002	NS	0.248	1.493	NS
Time to play (min)	0.022	0.129	NS	0.118	0.724	NS	0.337	2.090	*
Time to study (min)	0.127	0.769	NS	0.137	0.842	NS	-0.182	-1.080	NS
Time to watch TV & play videogame (min)	0.036	0.219	NS	0.078	0.474	NS	-0.078	-0.479	NS

r; Correlational coefficient, *t*; *t* value, *p*; *p* value, NS; not significant; *p < 0.05; **p < 0.01.

## Discussion

Investigations were done in 1998 and 2008 using go/no-go tasks to look into inhibitory control as an essential executive function implemented by the prefrontal cortex in children. The subjects in this study were all ordinary children and the conditions of their academic maturation were the same; the same kindergarten, primary school, and junior high school were used in both years.The number of errors for kindergarten groups was approximately the same for G1 but greater in 2008 for G2 and G3. The number of errors for primary school groups was virtually the same from G4 to G9. Comparisons between the results from 1998 and 2008 showed no statistically significant differences from G4 to G9. However, a change in the error peak patterns can be seen. The number of errors in 1998 peaks in G6, whereas the 2008 data show a maximum in G5 and a second increase in G8 with both groups showing a statistically significant increase over the same 1998 groups (Figure 3). In 2008, the number of errors was greater in G5 than G3, a pattern that was not observed in 1998. The second peak (G8) in 2008 was not seen previously. These changes indicate that during the 10 years between tests, the inhibitory control ability has declined.

The period from early childhood to school age is important for the development of several brain functions. Dowsett et al. [11] reported that the conceptual understanding of inhibition responses is acquired by the age of 7 in typically developing children.

For junior high school students, in 1998 the number of errors decreased gradually from G10 to G12. However, in 2008, the number peaked in G11. According to the statistical comparisons between 1998 and 2008, the number of errors in G10 and G11 increased significantly in 2008. In addition, the number of errors in G11 increased at a greater rate compared to 1998. This increase suggests diminished inhibition control ability in G11. This result shows diminished cognitive components, besides response inhibition, of the brain of Japanese children for the ten years from 1998 to 2008. We do not yet understand the cause, but it may be some kind of influence such as environmental changes.

Tamm et al. [12] reported that the go/no-go task requires multiple executive functions, including working memory, interference avoidance, and response withholding, which have been established as prepotent responses. Working memory provides for the need to access and manipulate information in the short-term memory system [13]. In recent years, the working memory model has been further supported by neuroimaging studies. Selective attention, the capacity to focus on one stream of information while shutting out irrelevant material, is needed to solve a task immediately [14]. Furthermore, the capacity to switch attention from one source to another is also necessary. These important roles are assumed by working memory. Almost all measures of short-term memory show a steady increase from the preschool years through adolescence: Behavioral measures of working memory systems improve substantially between the ages of 4 and 15 [15,16]. Based on this data, an increased error rate for go/no-go tasks reflects a changed condition in the inhibitory function that is an essential executive function implemented for working memory by the prefrontal cortex.

We carried out a survey to explore possible correlations that may be found in children’s lifestyle changes. Significant correlations between the number of errors in the go/no-go tasks and the hours spent watching television and playing videogames, bedtime, and the number of hours of sleep were found in some grades. Overall, however, no significant correlation between the number of errors in the go/no-go tasks and the lifestyle survey was found. From this, we conjecture that the change condition in the inhibitory function is not directly related to lifestyle factors such as the time you, wakeup, bedtime, hours spent studying or hours spent watching television and playing video games. Thus, other potential causes for these delays must be explored.

The Japanese lifestyle survey from 1956 to 1978 reported large time changes on the average [17]. Play -time decreased to 53 minutes, TV watching and listening to stereo and radio increased to 59 minutes, time doing housework decreased to 74 minutes, and studying increased to 104 minutes. This may be related to the decrease in physical activity. In the future, our studies need to include these things.

## Competing interests

The authors declare that they have no competing interests.

## Authors’ contributions

KT, OS and TM participated in the design of this study and drafted manuscript. HT, AY, KS and ST carried out data collection and analysed the data. HT provided the information about the psychological variables. ST carried out back translation into English, and confirmed phrasing nuances with HY and KS. OS, TM and HT evaluated the results of the study and reviewed the manuscript. All authors read and approved the final manuscript.
